# Silicone/Ag@SiO_2_ core–shell nanocomposite as a self-cleaning antifouling coating material†

**DOI:** 10.1039/c8ra00351c

**Published:** 2018-03-08

**Authors:** Mohamed S. Selim, Hui Yang, Feng Q. Wang, Xue Li, Yong Huang, Nesreen A. Fatthallah

**Affiliations:** Technical Institute of Physics and Chemistry, Chinese Academy of Science 29 Zhongguancun East Road, Haidian District Beijing 100190 China wangfq@mail.ipc.ac.cn yhuang@mail.ipc.ac.cn; Petroleum Application Department, Egyptian Petroleum Research Institute Nasr City 11727 Cairo Egypt; Shandong Provincial Key Laboratory of Fluorine Chemistry and Chemical Materials, School of Chemistry and Chemical Engineering, University of Jinan 336 West Road of Nan Xinzhuang Jinan 250022 China lixue0312@yahoo.com; Processes Development Department, EPRI Nasr City 11727 Cairo Egypt

## Abstract

The effects of Ag@SiO_2_ core–shell nanofiller dispersion and micro-nano binary structure on the self-cleaning and fouling release (FR) in the modelled silicone nano-paints were studied. An ultrahydrophobic polydimethylsiloxane/Ag@SiO_2_ core–shell nanocomposite was prepared as an antifouling coating material. Ag@SiO_2_ core–shell nanospheres with 60 nm average size and a preferential {111} growth direction were prepared *via* a facile solvothermal and a modified Stöber methods with a controlled shell thickness. Ag@SiO_2_ core–shell nanofillers were inserted in the silicone composite surface *via* solution casting technique. A simple hydrosilation curing mechanism was used to cure the surface coating. Different concentrations of nanofillers were incorporated in the PDMS matrix for studying the structure–property relationship. Water contact angle (WCA) and surface free energy determinations as well as atomic force microscopy and scanning electron microscope were used to investigate the surface self-cleaning properties of the nanocomposites. Mechanical and physical properties were assessed as durability parameters. A comparable study was carried out between silicone/spherical Ag@SiO_2_ core–shell nanocomposites and other commercial FR coatings. Selected micro-foulants were used for biological and antifouling assessments up to 28 days. Well-distributed Ag@SiO_2_ core–shell (0.5 wt%) exhibited the preferable self-cleaning with WCA of 156° and surface free energy of 11.15 mN m^−1^.

## Abstract 2

The effects of Ag@SiO_2_ core–shell nanofiller dispersion and micro-nano binary structure on the fouling release of silicone paints were studied. An ultrahydrophobic PDMS/Ag@SiO_2_ core–shell nanocomposite was prepared as an antifouling coating material.

## Graphical abstract

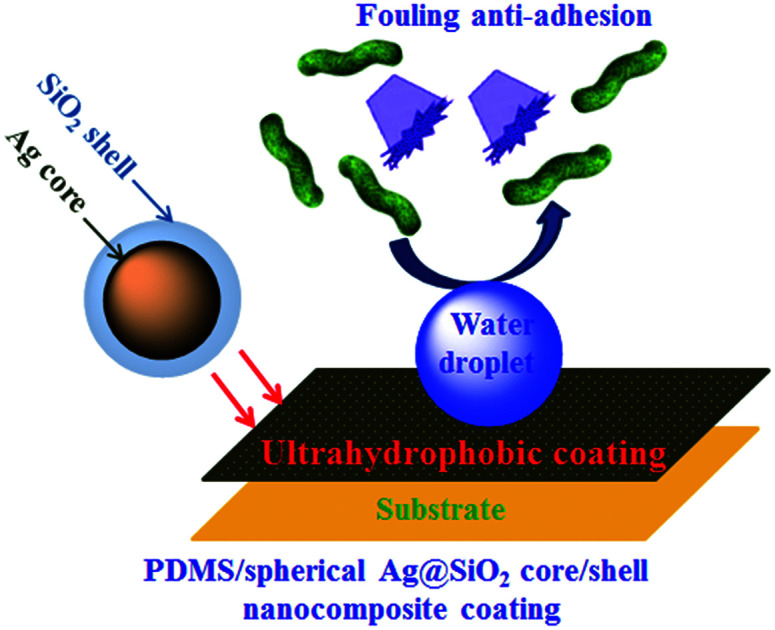

## 1. Introduction

Biofouling on ship hulls increases drag resistance and hydrodynamic weight which result in reducing shipping velocity and increasing fuel consumption and emissions of harmful air pollutants.^1,2^ Antifouling coatings based on organotin compounds pose a threat to the marine environment. Alternative tin-free antifouling coatings employing copper and/or booster biocides are the principal replacement coatings but these materials are also deleterious to the environment. Their toxic effects have been found to extend to non-target species with an ecological risk to 95% of organisms in the water column even at very low concentrations.^3^ The substantial environmental toxicity issues and the increased global restrictions on the applications of biocidal antifouling paints have motivated research in an eco-friendly way focusing on natural marine compounds and non-stick silicone fouling release (FR) coatings.^4^ Natural antifouling compounds face the same regulatory hurdles with the estimated cost of assembling data packages on efficacy and environmental fate and affect many millions of dollars, and the timeline for the approval process.^5^

Non-stick, silicone FR paints especially polydimethylsiloxane (PDMS) rely on a technology that can: (1) inhibit fouling settlements, (2) weaken fouling adhesion strength; *via* providing low friction and super-smooth surface.^6^ PDMS possess several advantages including feasibility, cost-effective, non-leachant properties of any toxicants, low porosity, stability in water, low surface energy, high thermal stability, ultra-high molecular mobility, repellency against fouling and high UV and oxygen resistance.^7^ Ultrahydrophobic surfaces with water contact angle (WCA) > 150° and low-contact-angle hysteresis of <5°, are effective self-cleaning materials.^8^ Innovation of organic/inorganic hybrid nanocomposites is a modern strategy for superior FR coating.^9,10^

Recently, core–shell nanostructured materials have received great interest in the fields of nanocomposite surfaces.^11^ The Ag core@SiO_2_ shell nanoparticles (NPs) are more interesting because of their typical unique chemical and physical properties.^12^ They are potentially used in various fields including antibacterial, anticorrosion and environmental applications.^13,14^ Several studies have highlighted the anti-fungal, antiviral and antifouling activities of Ag NPs.^15,16^ As a noble metal, Ag NPs have been widely used as an effective antimicrobial agent against bacteria, fungi, and viruses. Nano-Ag is less expensive and presents excellent antibacterial property compared with nanogold.^17^ Ag NPs are more efficient than Cu NPs against *Escherichia coli* and *S. aureus*.^18^ Among various antibacterial agents, Ag NPs are highly favorable because of their high toxicity to a broad spectrum of microorganisms but low cytotoxicity to higher animals.^19^ The high surface-area-to-volume ratio of NPs contributes to their unique physical, chemical, mechanical, and quantum size effect properties. Higher antibacterial properties are caused by increased {111} crystal planes.^20^ The polar properties of edged Ag spheres with densely packed {111} lattice plane, which exhibits the lowest surface energy per unit area and stability over other Ag nanostructures (cubic, wire, and triangular), which contain few {111} planes can afford a coating material with a high antifouling properties.^15,20^ Also, the hydrophobicity of a coated film is enhanced by insertion of Ag NPs.^21^

Silica is widely used as a stable coating for metal NPs, allowing the formation of stable nanostructures.^22^ Hybrid Ag core@SiO_2_ shell nanofiller structure combines the properties of two phases with varied chemical composition and crystal structure.^23^ SiO_2_ shell can increase the colloidal stability and dominate the distance between core particles within assemblies *via* shell thickness for various applications.^24^ Nano-silica shells are suitable for bio-conjugations because of their surprising surface properties.^25,26^

Pan *et al.*, reported the preparation of polyvinylidene fluoride–Ag/SiO_2_ nanocomposite membrane with antibacterial and antifouling properties.^27^ Le *et al.*, reported that 1 wt% Ag/SiO_2_ NPs in acrylic coating exhibit better antimicrobial corrosion activity than that of conventional 40 wt% Cu_2_O biocides.^28^ Huang *et al.*, reported the fabrication of Ag–SiO_2_/polyethersulfone membrane with high magical anti-bacterial and anti-biofouling properties.^29^ However no data were reported for the fabrication of silicone/Ag@SiO_2_ core–shell based nanocomposites for marine antifouling coating.

In the present study, an eco-friendly series of silicone/Ag@SiO_2_ core–shell hybrid composites was fabricated for shipping industry. Silver nanospheres were successfully synthesized *via* solvothermal method in a short reaction time. A controlled SiO_2_ shell (2–5 nm thickness) was formed using a modified Stöber method by dominating the silica precursor concentration.

Solution casting method of silicone/Ag@SiO_2_ core–shell grown in {111} direction was achieved, resulting in ultrahydrophobic self-cleaning and low surface free energy (SFE). Different nanofiller percentages were incorporated in the silicone matrix to study the structure–property relationship. The surface non-wettability was studied *via* WCA, SFE and atomic force microscopy (AFM) measurements. The mechanical and physical characteristics of the coated specimens were also assessed by using different techniques. Biodegradability evaluation and turbidimetric prediction was applied to trace concentration and mass of bacterial suspensions. The designed nanocomposite is potentially useful as an environmental, ultrahydrophobic FR and self-cleaning coating material of ship hull.

## 2. Materials and methods

### 2.1. Materials

Silver nitrate (AgNO_3_, 99%), tetraethyl orthosilicate (TEOS, Si(OC_2_H_5_)_4_, 98%) polyvinylpyrrolidone (PVP, *M*_w_ 40 000), ethylene glycol ((CH_2_OH)_2_, 99%), octamethylcyclotetrasiloxane (D4, [–Si(CH_3_)_2_O–]_4_, 98%), platinum(0)-1,3-divinyl-1,1,3,3-tetramethyldi-siloxane complex solution in xylene known as Karstedt catalyst (platinum ∼2%), 1,3-divinyltetramethyldisiloxane (C_8_H_18_OSi_2_, 97%), poly(methyl siloxane) (PMHS; number average molecular weight (*M*_n_) = 1700–3200), 98%) and ammonia solution (NH_4_OH, 28–30%) were all purchased from Sigma-Aldrich Chemical Co. Ltd., USA. Potassium hydroxide (KOH, 98%), anhydrous ethanol (AR), acetone was delivered from Acros Company (Belgium).

### 2.2. Growth of Ag@SiO_2_ core–shell NPs

Silver nanospheres with 60 nm average size were synthesized *via* a high-temperature solvothermal method by using ethylene glycol. In brief, AgNO_3_ (0.25 g) and PVP (1.25 g) were dispersed in 100 mL of ethylene glycol. This solution was heated to 130 °C for 20 min under vigorous stirring and continued for 1 h without further stirring. Then, 400 mL of acetone was added followed by sonication and centrifugation to separate the prepared Ag NPs from the ethylene glycol. The precipitated NPs were sonicated in ethanol (5 mL) to form 0.02 g mL^−1^ of Ag NPs/ethanol solution.

A modified Stöber method was used to prepare Ag@SiO_2_ core–shell nanospheres (2–5 nm thickness of SiO_2_) as follow:

5 mL of Ag NPs/ethanol solution (0.02 g mL^−1^) obtained above was ultra-sonicated in 80 mL ethanol and stirred for 20 min at 600 rpm at room temperature (RT), followed by adding 20 mL distilled water and 1.2 mL ammonium hydroxide solution to the mixture. Then, 15 μL TEOS previously dissolved in 5 mL ethanol was introduced drop by drop with continuous stirring, and the reaction was continued for 12 h. The Ag@SiO_2_ core–shell NPs were washed with a mixture of distilled water and ethanol for 3 times and finally sonicated in 10 mL ethanol.

### 2.3. Ultrahydrophobic nano-coating design

**Scheme 1:**
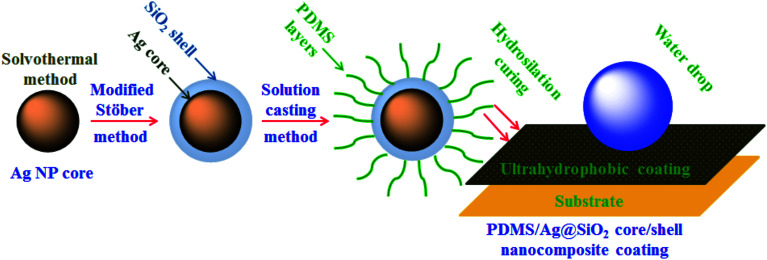
Solution casting of PDMS/spherical Ag@SiO_2_ core–shell nanocomposites and film formation *via* hydrosilation curing.

Vinyl ended PDMS with ultra-high MW was prepared *via* chain growth polymerization of D_4_. In a typical polymerization reaction, 30 g of D_4_ (after vaccum distillation) and 0.18 g of CsOH were added into a three neck flask. The polymerization reaction was performed at 130 ± 5 °C with vigorous stirring under a nitrogen atmosphere for 2.5 h. Divinyltetramethylsiloxane (4 × 10^−4^ mol) was added to the mixture for end capping and reacted for another 3 h. Then the reaction temperature was lowered slowly to RT and left overnight under stirring to allow chain termination. After the unreacted monomers were removed by vacuum distillation, orthophosphoric acid was used for neutralization followed by filtration. Solution casting approach was applied to prepare a series of PDMS/Ag@SiO_2_ core–shell nanocomposites by dispersing nanofiller with different concentrations (0.05–3 wt%) in the prepared PDMS (Scheme 1).

Unfilled silicone and Ag@SiO_2_ core–shell filled PDMS nanocomposites were cured by hydrosilation curing mechanism^30^ as follow:

Solution A was formed by adding 0.1 g of Karstedt catalyst solubilized in 30 mL of trichloroethylene to a flask containing 25 g of PDMS and 60 mL methylbenzene. Meanwhile, 0.6 g of PMHS in 20 mL methyl benzene represented solution B. Under vigorous stirring, solution B was added to solution A and the resultant mixture was degassed for 15 min. The degassed solution was ready to apply on different substrates and was left to cure for 12 h at RT.

### 2.4. Analysis

Crystallinity of NPs was tested *via* X-ray diffraction (XRD) using a PANalytical X'Pert PRO diffractometer (Netherlands). The diffraction patterns were determined *via* CuKα radiation, 2*θ* angle of 30–80°, and interatomic spacing of 1.23–2.82 Å. The size distribution profile of Ag@SiO_2_ core–shell was performed by Brookhaven 90Plus particle size analyzer (90Plus, United States). Morphology and particle size of Ag@SiO_2_ core–shell were determined by JEM-2100F Field emission transmission electron microscope (FETEM) (JEOL, Japan) at 200 kV. The core–shell NPs were dispersed in ethyl alcohol, and two drops of the solution were put onto carbon-coated TEM grids prior to image capture.

The crystal structure and lattice planes identification was studied by selected area electron diffraction (SAED) analysis. The silicone/Ag@SiO_2_ core–shell nanocomposite was sectioned to 150 nm thick by an ultracryomicrotome (Leica Ultracut UCT, Austria) with a sharp diamond cryoknife at −150 °C. Elemental composition of Ag@SiO_2_ core–shell NPs was analyzed using EDS (X-Max 50, Oxford Instruments, USA) at 30 kV. Shape and surface topology were observed by a FESEM (JEOL JSM530, Japan) at 30 keV. The Ag@SiO_2_ core–shell sample was sonicated in ethanol, and two drops of the solution were mounted on a glass slide and air dried. In order to avoid the charging effect under the electron beam, the specimen was spatter-coated with gold. FTIR analysis was conducted using a spectrometer (Thermo-Fischer Nicolet™ iS™10, United States). The scan region and spectral resolution were 500–4000 cm^−1^ and 0.5 cm^−1^, respectively.

### 2.5. Surface properties

WCA and SFE of the coated specimens were determined to assess the non-wettability properties. Static WCA was measured by a contact angle goniometer (Krüss GmbH, Germany) by sessile bubble method. The dynamic CA determinations including advancing and receding angles were evaluated by increasing/decreasing liquid method. CA hysteresis which expresses the difference between advancing and receding CAs is essential to investigate the surface heterogeneity.

The specific surface area (*S*_BET_) of the nanocomposites was determined by low-temperature (77.4 K) nitrogen adsorption–desorption isotherms which were recorded using a Sorptometer KELVIN 1042 (COSTECH Instruments) adsorption analyzer. Samples were previously outgassed at 473 K for several hours. The specific surface area (*S*_BET_) was calculated using the Brunauer–Emmet–Teller (BET) method.^31^

Total SFE (*γ*^total^_S_) of the coated films was calculated *via* geometric mean method based on Owens, Wendt, Rabel, and Kaelble (OWRK) technique.^32^ This technique proposed that *γ*^total^_S_ is the sum of dispersive and polar components. It can be calculated by measuring the contact angle (*θ*) value for each surface using two different solvents such as water and diiodomethane as illustrated in (eqn (1) and (2)):^33,34^1
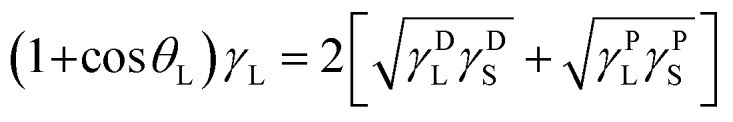

2 *γ*^total^_S_ = *γ*^D^_S_ + *γ*^P^_S_

where *θ*_L_ and *γ*_L_ represent the contact angle and the surface tension of the testing liquid. *γ*^D^_L_ and *γ*^P^_L_ are the dispersion and polar surface tension of the liquids used, while *γ*^D^_S_ and *γ*^P^_S_ are the dispersion and polar surface tension of the applied surfaces, respectively.

The surface topography of the unfilled PDMS and silicone/Ag@SiO_2_ core–shell nanocomposite coatings was elucidated by an atomic force microscope (AFM, XE7, Park Systems Co., Ltd. South Korea) at ambient conditions. The root mean square roughness (RMS) of the coated samples was assessed by AFM apparatus software XEL. The measurement was set at a resonance frequency of 300 kHz, a scan rate of 0.7 Hz and a force constant of 40 N m^−1^.

### 2.6. Physico-mechanical investigation

Viscoelastic characteristics of the painted films were determined by using a Triton dynamic mechanical analysis (DMA, UK) instrument following ASTM412. Testing the rectangular samples was performed in tension mode by a single frequency at 25 °C, instrument preload of 0.1 N and 1 to 27 mm amplitude. The coating's elasticity and adherence were investigated *via* three mechanical tests known as impact, crosshatch and bend tests. Mild steel specimens (17 cm × 9 cm × 0.08 cm) were used for mechanical tests. A two component epoxy paint (mixing ratio 3.7 : 1 by weight) was applied as a primer layer. The tie coat was formed of two components of silicon/epoxy hybrid paint (mixing ratio 4 : 1 by weight) as tie paint. The final layer of silicone/Ag@SiO_2_ core–shell nanocomposite coating was stretched with a dry film thickness of 150 μm. The resistance against damage was tested by using Sheen tubular impact tester (Model Ref BG5546, UK) *via* dropping weight (1000 g) (ASTM D2794-04). Sheen crosscut tester (model SH 750, UK) was used to determine the coating-substrate bonding strength using a cutter of steel with 1.5 mm × 6 teeth. According to ASTM D3359, a pressure-sensitive adhesive tape was attached over the cut, smoothed and pulled. Paint formability was checked *via* the test of mandrel bending (ASTM D522). Sheen bending tester model Ref. 809 (UK) was applied in the mandrel diameter range from 3.1 : 38 mm.

### 2.7. Biological studies

#### 2.7.1. Microorganisms' details

Microfoulants of bacteria and fungi organisms that cause marine fouling were selected to investigate the FR behaviour of the coated specimens. Bacillus subtilis and *Staphylococcus aureus* (Gram-positive bacteria), *Pseudomonas aeruginosa* and *Escherichia coli* (Gram negative bacteria), molds of *Candida albicans* (yeast) and *Aspergillus niger* (fungus) were used in the biological tests because of their strong fouling activity. They were widely used to assess the biofilm formation on different surfaces.^35–38^ These strains were delivered from MIRCEN, Egypt and the microbial growth is permitted in a medium of nutrient-infused liquid for 28 days at 35 °C.^39^

#### 2.7.2. Biodegradability test

Biodegradability percentage (% BD) of the tested specimens was determined under aerobic condition from the weight (*W*) difference between control and standard samples as stated in eqn (3).^38^

3 % BD = ([*W*_Control_ − *W*_Standard_]/*W*_Standard_) × 100

#### 2.7.3. Growth measurements

The turbidity was determined as absorbance in optical density measurements (density/mL) using ATI Unicam 8625 Ultraviolet/Visible spectrophotometer at 600 nm for bacteria & yeast and at 700 nm for fungi. Desired concentrations of bacterial suspensions were standardized adjusting initial optical density (O.D) as 0.02, 0.1 and 0.5 nm for bacteria, yeast and fungi, respectively. The stationary phase of the growth curve of each of the tested organisms to avoid cell size variation is expressed as O.Ds. Since wavelengths between 500–600 nm and greater can express the bacterial cell numbers whereas a wavelength of 700 nm or greater would reduce the absorption effects due to pigments of fungal spores.^40^ Non-injected broth medium is considered to be control O.D. Optical densities were recorded at constant intervals in the whole time experiment. The viable cells percentage was calculated using eqn (4) after incubation in different bacterial, yeasts and fungi strains:^41^

4 % Viable cells (*I*) = O.D_T_ × 100/O.D_C_

where, O.D_T_ and O.D_C_ represent the optical dentistry of the tested specimen and the control, respectively. In this approach, cell number increases directly as the growth increases leading to proportional increase in the optical density of the medium.

#### 2.7.4. Polarized optical microscope (POM)

POM images were used to elucidate the biofilm coverage on the coated specimens after insertion in the microfouling medium. The images were captures by model BHS, Olympus Microscope (Japan).

## 3. Results

### 3.1. Characterization of Ag@SiO_2_ core–shell nanospheres

**Fig. 1:**
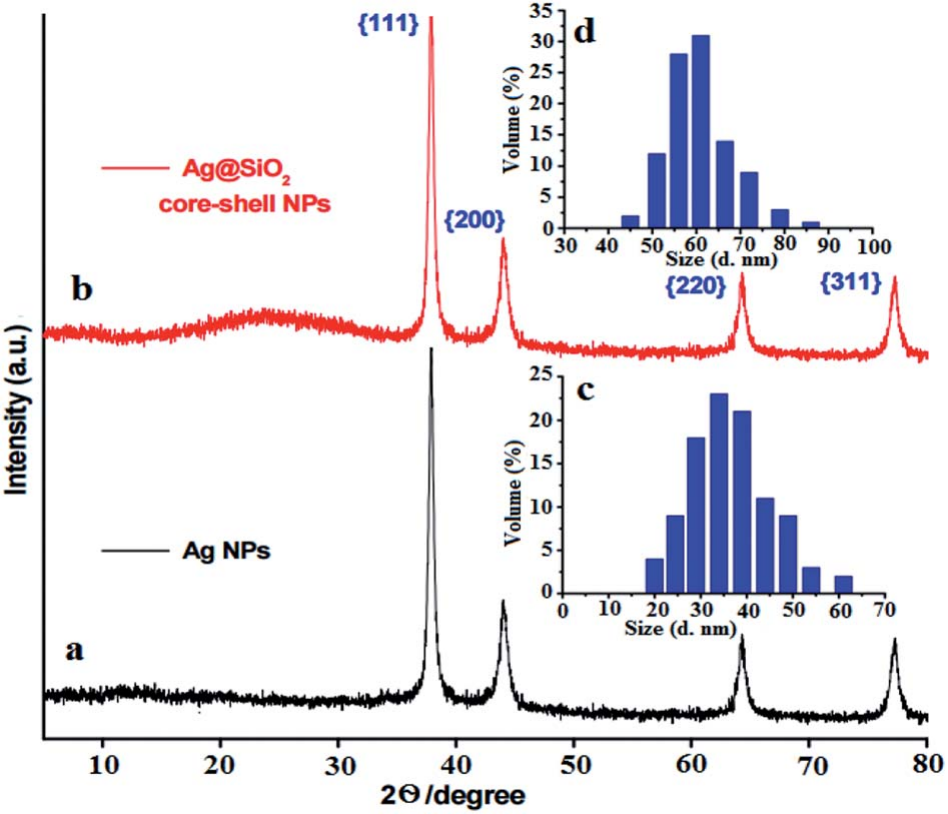
(a) XRD pattern of the prepared (a) Ag NPs and (b) Ag@SiO_2_ core–shell nanospheres and inside the DLS of (c) the prepared Ag NPs and (d) Ag@SiO_2_ core–shell nanospheres.

**Fig. 2:**
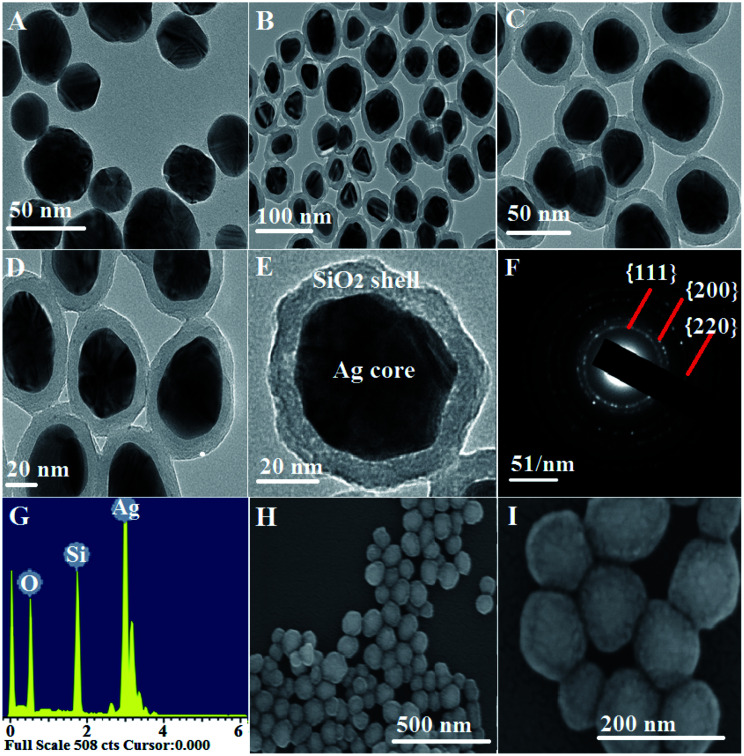
(A) Is the TEM image of the prepared Ag core NPs; (B–D) are the TEM images of the prepared Ag@SiO_2_ core–shell nanospheres at different magnifications; (E) TEM images of the core–shell structure and indicate the controlled SiO_2_ shell thickness to be 2–5 nm; (F) corresponding to SAED patterns of the as-synthesized Ag@SiO_2_ core–shell NPs which goes in agreement with the results from XRD; (G) EDS image of the prepared Ag@SiO_2_ core–shell NPs; and (H and I) are the corresponding FESEM images of the prepared Ag@SiO_2_ core–shell nanospheres.

A facile preparation of Ag nanospheres was successfully conducted within a short reaction time. TEOS concentration is the main factor to control the thickness of SiO_2_ shell. The crystalline information and morphological homogeneity of the Ag@SiO_2_ core–shell NPs were obtained using XRD (Fig. 1). The prepared Ag crystals exhibit sharp diffraction peaks at 2*θ* values of 37.821, 44.321, 64.231 and 77.421 corresponds to {111}, {200}, {220} and {311} lattice planes (Fig. 1a). No remarkable silica peaks were observed for the Ag@SiO_2_ core–shell particles which indicate that silica shell is amorphous in nature (Fig. 1b). The crystal plane {111} facet is more intense because of its predominant orientation than the other peaks. Debye–Scherer equation was used to determine the average size of Ag@SiO_2_ NPs (as indicated in eqn (5))^42,43^ which was indicated to be 60 nm.5
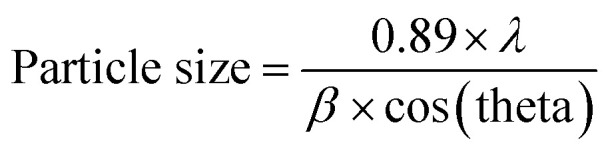
where the X-ray radiation is expressed in *λ*, and *β* and *θ* are the line broadening at half of the maximum intensity and the measured Bragg's angle. Fig. 1c and d indicate that the DLS analysis of Ag and Ag@SiO_2_ core–shell NPs. The average diameter of Ag NPs is about 40 nm, and it increased to about 60 nm after coating thickness of 2–5 nm SiO_2_ interlayer. FTIR spectrum of Ag@SiO_2_ core–shell NPs (ESI, Fig. S1†) indicate that the absorption peaks at 798 and 960–1103 cm^−1^ are related to the symmetric and asymmetric vibration of Si–O–Si from silica shell, respectively. Absorption at 1638 cm^−1^ and 3359 cm^−1^ corresponds to O–H stretching vibration from water and ethanol respectively.^44,45^ TEM pictures of the prepared Ag NPs are expressed in Fig. 2A. Well-dispersed Ag@SiO_2_ core–shell nanospheres are indicated in Fig. 2B–E with uniform 2–5 nm SiO_2_ shell thickness. Overall, the prepared nanospheres presented a 60 nm average diameter, and a single crystal structure without agglomeration. TEM observation confirms that SiO_2_ covered Ag NPs. The crystallinity of Ag NPs was further confirmed *via* SAED (Fig. 2F). Distinct ring patterns were monitored, and the crystal facets of {111}, {200}, and {220} were indexed to approve the NPs' polycrystalline structure. The findings indicated that the main facet is the {111} crystal plane, which may represent the desired self-cleaning antifouling characteristics such as low surface energy, antibacterial activity, and nearest neighbour atoms per unit area. Fig. 2G illustrates the elemental map of the Ag@SiO_2_ core–shell nanospheres *via* the EDS spectrum. The analysis indicated the presence of Ag, Si and O elements without impurities. The sample provided the following sample content results: 53.86%, 23.65%, and 22.49% for Ag, O, and Si elements by weight, and their atomic percentage was 32.49%, 39.41%, and 28.1%, respectively. The SEM pictures of Ag@SiO_2_ NPs Fig. 2H and I reflect the well-dispersed nanospheres with super-smooth and homogenous surface nature.

### 3.2. Nanocomposite film formation

FTIR spectrum of the prepared vinyl ended PDMS (ESI, Fig. S1†) indicated that the absorption bands observed at 2969 and 2910 cm^−1^ refer to the asymmetric –CH_3_ stretching. The bands observed at 1595, 1417, 1263, 1099, 801, and 702 cm^−1^ refer to Si–CH=CH_2_ stretching absorption, –CH_3_ symmetric deformation, CH_3_ symmetric deformation, Si–O–Si asymmetric deformation, Si–O–Si skeletal stretching, and symmetric stretching of the Si–C bond in –Si (CH_3_) group, respectively. The absence of any absorption peak at 2060 cm^−1^ and 3000 cm^−1^ to 3500 cm^−1^ confirmed successful synthesis of pure vinyl-terminated PDMS without impurities. Unlike condensation-cured PDMS, hydrosilation-cured PDMS exhibits advanced FR properties for marine shipping, such as better stability and hydrophobicity in water. High *M*_W_ of vinyl terminated silicone nanocoatings afford ultra-high FR behavior with improved Young's modulus, tensile strength, elongation at break contrary to the low *M*_W_ analogues.^32^ Incorporation of Ag@SiO_2_ core–shell nanospheres in the silicone matrix exhibits improved FR properties. The newly developed silicone/Ag@SiO_2_ core–shell nanocomposites is an eco-friendly coating material for self-cleaning and antifouling applications.

**Fig. 3:**
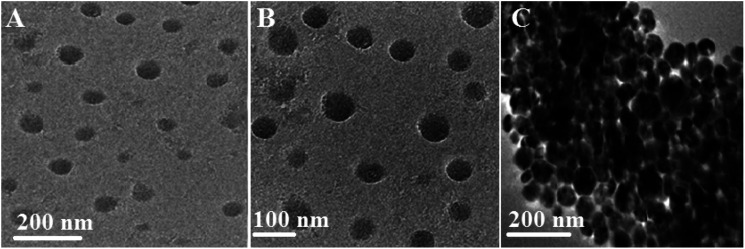
(A) and (B) corresponding TEM images of the as-synthesized well-dispersed PDMS/spherical Ag@SiO_2_ core–shell nanocomposites (0.5% nanofillers) at different magnifications and (C) is the TEM image of the prepared PDMS/spherical Ag@SiO_2_ core–shell nanocomposites at high filling concentration that cause agglomerations (3 wt% nanofillers) at different magnifications.

TEM was used to study the dispersion of Ag@SiO_2_ core–shell nanospheres in the silicone resin. The Ag@SiO_2_ core–shell NPs were indicated as dispersed dark spheres, and surrounded by uniformly bright PDMS background. TEM captures of PDMS/Ag@SiO_2_ core–shell composites (0.5% nanofiller) showed well-dispersion and no agglomeration characteristics (Fig. 3A and B). Excellent nanofiller dispersion increased NPs' surface area to the volume ratio, matrix–NPs interfacial bonding and thus improved self-cleaning FR behaviour. By contrast, higher filler percentages (up to 3 wt%) caused aggregation and clustering (Fig. 3C) that increased bonding strength of fouling organisms on the submerged surfaces.

### 3.3. Water repellent behavior

Ultrahydrophobic materials with smart surfaces and interfacial characteristics are important for fouling prevention due to the reversible dynamic variation in the film non-wettability and physico-mechanical properties. Ag@SiO_2_ core–shell nanospheres possess superior surface properties for applying in potential polymer brush systems for marine applications. The non-wettability characteristics were studied *via* WCA, SFE and AFM measurements.

**Fig. 4:**
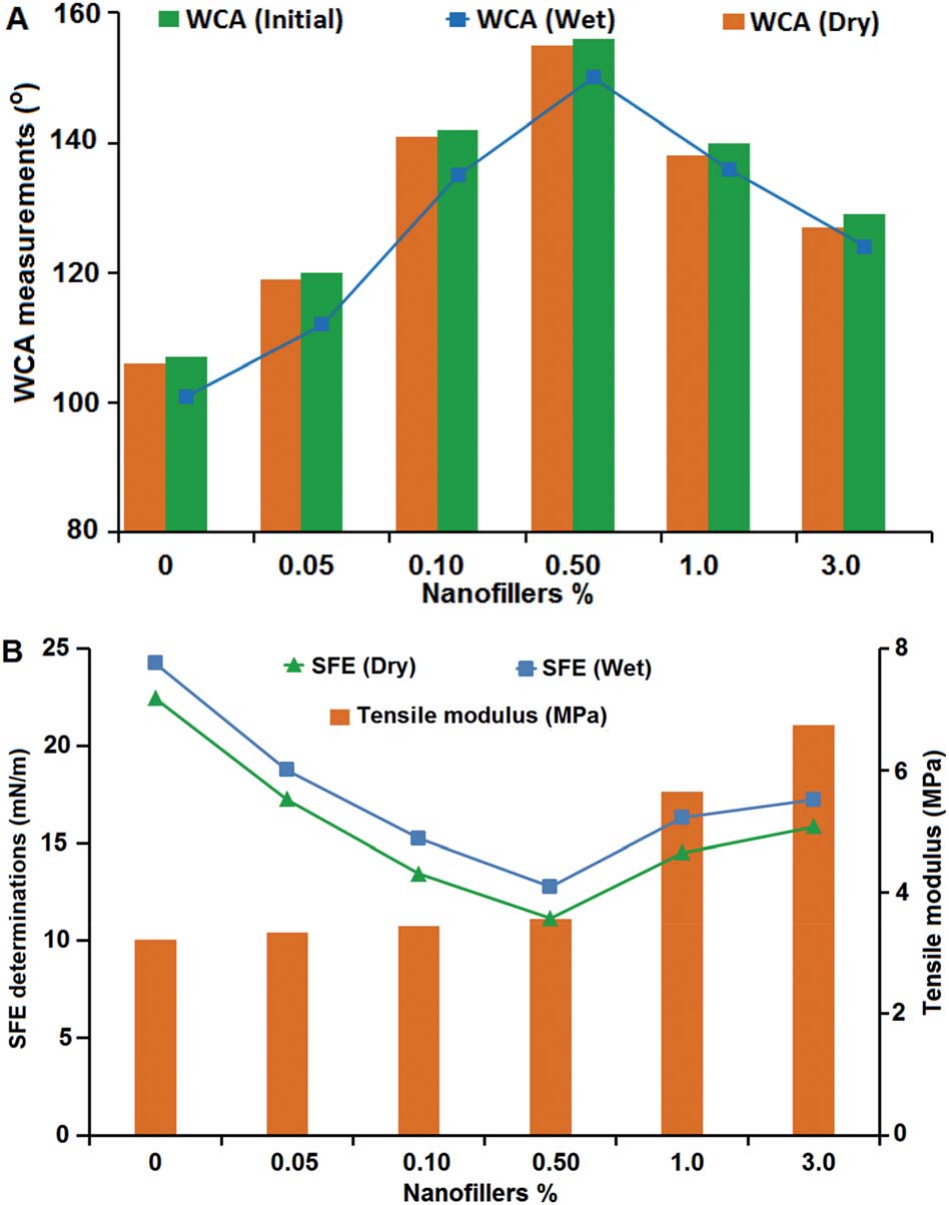
(A) WCA measurements of the virgin silicone and PDMS/Ag@SiO_2_ core–shell nanocomposites before and after immersion and also after drying (error bars represent ±2° standard deviations based on three different measurements) (B) SFE determinations of the virgin and PDMS/Ag@SiO_2_ core–shell nanocomposites before and after wet immersion in demineralized water for one week (error bars represent ±0.1 standard deviations based on three measurements) and inside tensile modulus values of the fabricated polymer and nanocomposites (error bars represent ±0.05 standard deviations from three replications).

Water-repellency characteristics of the coated specimens were studied through static WCA measurements before, after submerged in water and after drying (Fig. 4A). WCA measurement for the virgin PDMS coating was 107° ± 1°. WCA increased with insertion of different Ag@SiO_2_ core–shell NP concentration up to 0.5% (156° ± 3°). This ultrahydrophobic surface is produced by well dispersion of Ag@SiO_2_ core–shell nanospheres and improved polymer–NPs interfacial bonding. As a result, coating surface possesses super-smoothness and surface inertness, which can resist the adhesion of any pollutants or bacteria. Furthermore, the advancing and receding CAs were measured to investigate the surface hydrophobicity of the fabricated coatings (ESI, Fig. S2†). The results confirmed increasing the advancing and receding CA with well-dispersion of nanofiller (0.5 wt%). CA hysteresis, the difference between the advancing and the receding CAs, was also determined to confirm the surface non-wettability and chemical heterogeneity. The CA hysteresis of the virgin PDMS matrix (19.4°) was reduced with insertion of 0.5 wt% Ag@SiO_2_ core–shell nanofillers (6°) which indicated that the surfaces have predominant self-cleaning property. High WCA (>150°) and low CA hysteresis (<10°) are essential factors for ultrahydrophobic self-cleaning surfaces.^46^

By contrast, non-wettability showed a different behavior at higher Ag@SiO_2_ core–shell loadings; the WCA curve decreases inversely (from 1–3% nanofillers) and thus the self-cleaning reduced because of agglomeration. The static WCA decreased (to 129°) and the CA hysteresis increased (up to 13.1°) with core–shell filler insertion up to 3 wt%.

Particle clustering decreased the NPs' surface area and minimized polymer–NPs interfacial bonding; thus reduced the self-cleaning and FR ability. The values of *S*_BET_ of the silicone nanocomposites increased with nanofiller loading up to 0.5 wt% because of the increase surface area of the well-dispersed nanofillers, while decreased at higher concentrations because of the NP agglomeration (Fig. S3†). Our finding also indicates that WCA reaches to a value close to that obtained before immersion under drying condition. Therefore, the unfilled PDMS and silicone/Ag@SiO_2_ core–shell surfaces exhibit reversibly tunable characteristics.^47^

The SFE of the coated samples was studied before and after water submersion by using the geometric mean approach (Fig. 4B). The recorded values clarified that *γ*^total^_s_ was reduced gradually at lower nanofiller concentrations and ranged from 21.28 mN m^−1^ for virgin PDMS down to 11.15 mN m^−1^ for 0.5% Ag@SiO_2_ core–shell NPs.

The reduction in the SFE with incorporation of 0.5 wt% Ag@SiO_2_ nanospheres is a crucial effect of well-distribution of nanofillers in the PDMS chains. Well-dispersion of Ag@SiO_2_ orientation around {111} facets enabled a minimal interfacial energy surface that effectively affected the selective surface exposure properties, nearest neighbour atoms per unit area and chemical activity of the nanofiller coatings, leading to pronounced smoothness and FR efficiency. Fouling organisms cannot settle on such ultra-smooth, homogenous and self-cleaning surface and even their bonds with the coatings can be easily removed hydrodynamically.

On the other hand, *γ*^total^_s_ raised with further increasing nanofiller concentrations till 18.41 for 3% nanofillers. SFE increase at higher nanofiller loadings because of the NPs' agglomeration and particle clustering. The clustering and condensation of NPs over each other decrease NPs' surface area and the interfacial bonding with PDMS chains. NP interaction with each other caused by destabilizing effect of high nanofiller concentration in the matrix and van der Waals adhesion force between the particles increases. This clustered NP-matrix surface enable fouling organisms to settle easily as a result of reduced self-cleaning ability and increasing wetting characteristics and un-homogenous topology.

**Fig. 5:**
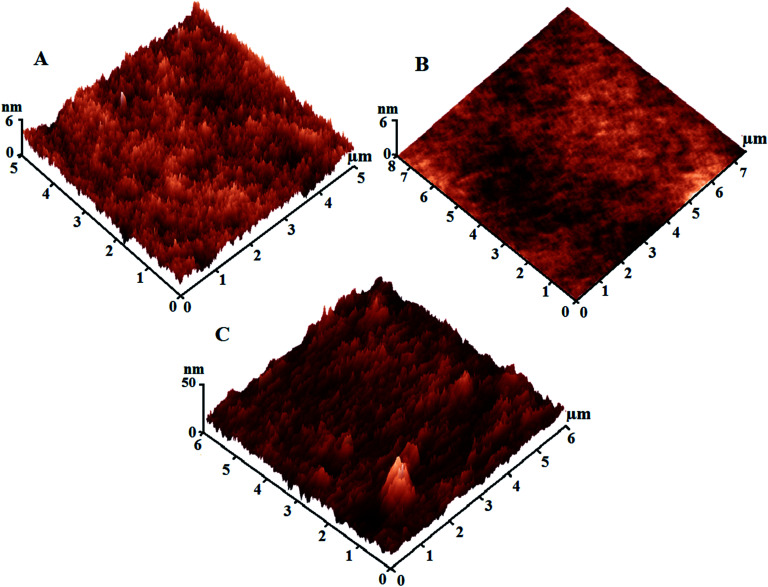
Three-dimensional AFM surface images of the prepared surfaces; (a) unfilled PDMS surface; (b) PDMS/spherical Ag@SiO_2_ core–shell nanocomposites (0.5% nanofillers); (c) PDMS/Ag@SiO_2_ core–shell nanocomposites (3% nanofillers).

Our findings indicated that the low SFE of the silicone/Ag@SiO_2_ core–shell nanofiller design is mainly accompanied by the high-density of {111} facets along the spherical, face-centered-cubic (fcc) Ag nanocrystal domains. This crystal plane is more powerful against fouling organisms than to other fcc planes.^48^ Small-sized NPs show high antibacterial properties against bacteria because of their {111} crystal planes.^15^ Silica shell can cause excellent dispersion and surface morphology stabilization of Ag NPs. Coatings' surface topology was tested by AFM test (Fig. 5). Unfilled PDMS film (Fig. 5A) reveals a uniform and featureless surface with RMS of 1.4 nm. With increasing the nanofiller concentrations up to 0.5 wt%, the topological homogeneity and surface smoothness increase with RMS of 0.88 nm (Fig. 5B). This is caused by the improved NPs' surface area, polymer–NPs interfacial bonding which can afford self-cleaning FR performance. High nanofiller concentrations up to 3% (Fig. 5C) in the silicone composites caused agglomeration and surface heterogeneity with RMS of 12.3 nm. Fouling organism can adhere easily on the clustered surfaces.^4,30^ Thus, lower fouling resistance was observed for agglomerated films.

### 3.4. A comparable study of the FR performance

Ultrahydrophobic self-cleaning properties were greatly affected by nanofiller enrichment. WCA and SFE were improved by well-dispersion of Ag@SiO_2_ core–shell nanofillers (0.5 wt%). Our developed FR coatings of PDMS/Ag@SiO_2_ core–shell nanocomposites were compared with other commercial and sounded coating surfaces, such as the following:

- Two commercially used antifouling models, namely, Sylgard 184 (hydrosilation-cured silicone surface) and RTV11 (condensation-cured silicone surface) from Dow Corning company products.^49^

- A developed Sylgard 184/sepiolite-MWCNT nano-coating system.^50^

- Tailored easy-cleaning hydrosilation-cured PDMS/Cu_2_O nanocube composites (with well-distributed 0.1 wt% cubic Cu_2_O nanofiller loadings).^51^

**Fig. 6:**
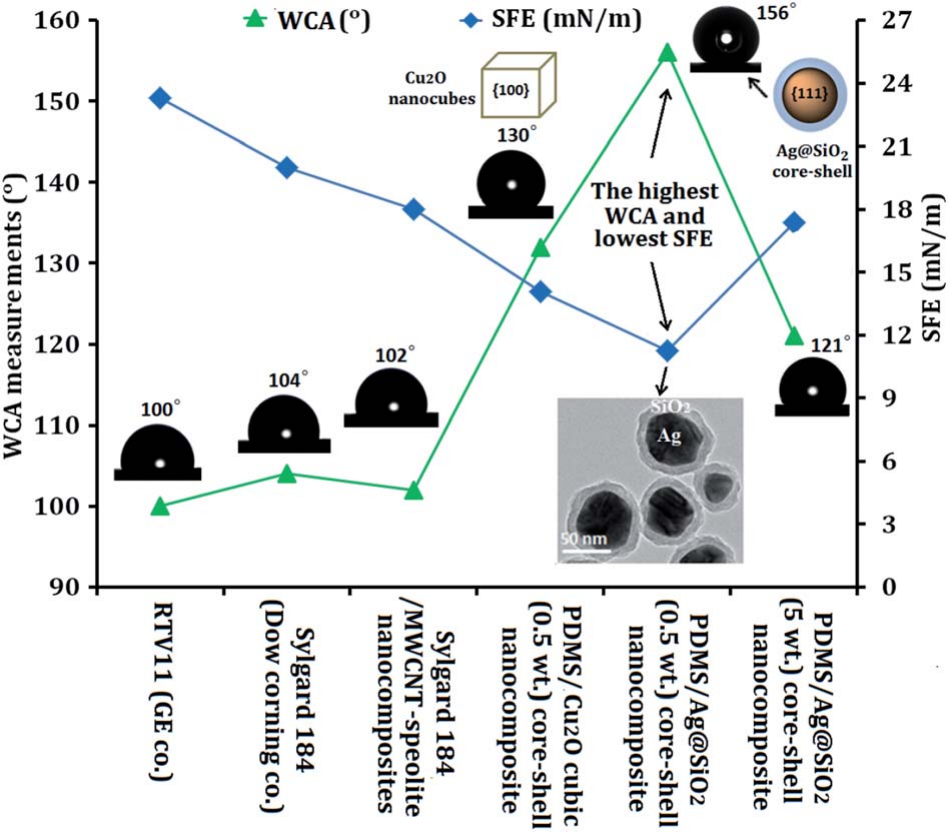
Comparable study of the FR and self-cleaning performance of PDMS/spherical Ag@SiO_2_ core–shell nanocomposites and other commercial developed and sounded FR surfaces.

Such nanocomposite surfaces were applied in previous studies for self-cleaning FR coatings (Fig. 6). A comparable study was carried out between the developed silicone/Ag@SiO_2_ nanocomposites and other commercial FR paints following the hypothesis of Wynne *et al.*,^49^ who compared Sylgard 184 and RTV11 by use of WCA and SFE measurements. Sylgard 184 exhibited higher hydrophobicity and stability in water than RTV11. Comparing the static WCA and SFE data of these two coatings showed that Sylgard®184 introduced higher contact angle (104°) than RTV11 coating (100°).^52^ Also, the SFE of Sylgard® 184 (20 mN m^−1^) was lower than RTV11 (approximately 23.3 mN m^−1^). Thus, Sylgard® 184 was more effective in resisting fouling adhesion than RTV11. Sylgard® 184 was modified with multi-wall carbon nanotubes (MWCNT) (up to 0.2% nanofillers) and sepiolite (from 0 to 10%) to enhance SFE and FR properties. After modification, the SFE decreased to 18 mN m^−1^ but the WCA only slightly changed. The filled coatings were more hydrophobic than the unfilled ones, and thus presented higher tendency to retard fouling.^53^ The previously tailored PDMS/Cu_2_O (0.1 wt% nanocubes) composites showed higher FR performance compared with RTV11 and modified Sylgard®184; the WCA increased up to 130°, and the SFE decreased to 14.1 mN m^−1^.

In current work, the fabricated silicone/Ag@SiO_2_ (0.5 wt%) core–shell nanocomposites exhibited the maximum WCA (156°) and minimum SFE (11.15 mN m^−1^) than the mentioned commercial or previously reported coatings. Also, this nanocomposite showed ultra-smooth topology as indicated in the AFM results obtained from well dispersion of Ag@SiO_2_ nanofillers with a preferential {111} growth direction in the PDMS matrix. This improved the surface self-cleaning and weakened the surface–fouling bonds to give fouling inertness. This reflected that {111} facets of spherical Ag@SiO_2_ core–shell enabled a minimal SFE and fouling adhesion than {100} facets of Cu_2_O nanocubes. These FR results are more prominent than agglomerated nanocomposites (5 wt% nanofillers) which exhibited WCA of 121° and SFE of 17.36 mN m^−1^. The agglomerated nanocomposite film showed reduced hydrophobicity due to the high surface polarity which makes the water to fill the grooves very easily through capillary action.

### 3.5. Antifouling assessments

**Fig. 7:**
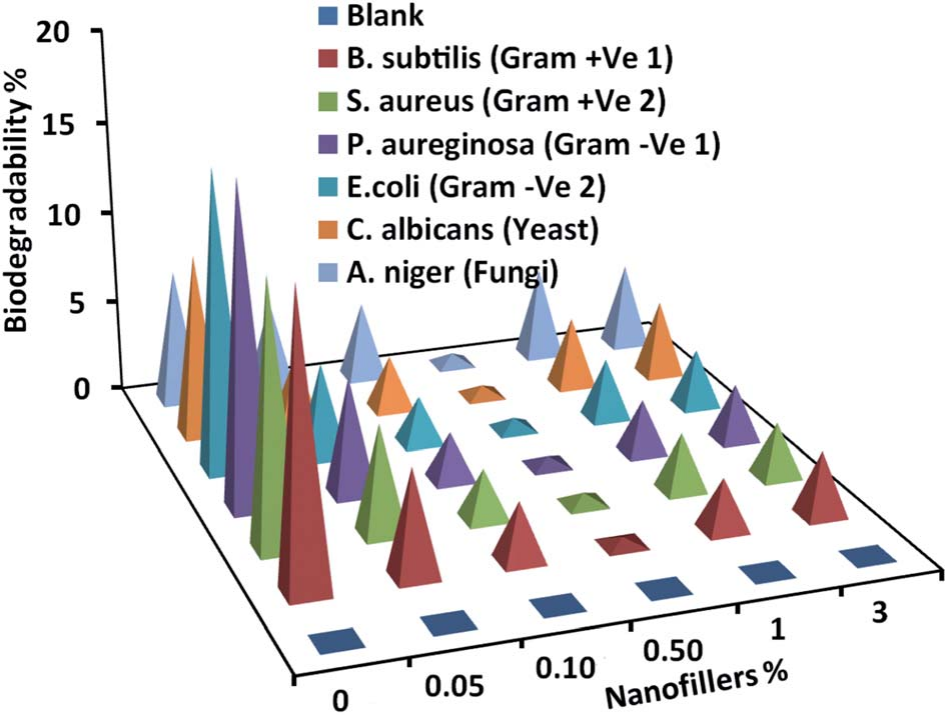
Biodegradability determinations of the virgin silicone and PDMS/spherical Ag@SiO_2_ core–shell nanocomposites against different micro-foulants.

Testing the biodegradability percentage of the coated specimens by selected micro-foulants is necessary to assess fouling anti-adhesion behavior. Unfilled and filled PDMS nanocomposite coatings were exposed to the selected micro-organisms' medium for 28 days and the outcomes were reported in Fig. 7. The biodegradability percentage decreased gradually with loading of Ag@SiO_2_ core–shell nanospheres up to 0.5 wt%. The excellent surface inertness reflected the self-cleaning and FR performance obtained by well-dispersion of Ag@SiO_2_ nanofillers. This is the result of increasing NP's surface area and their interfacial bonding with the polymer matrix. On contrarily, biodegradability percentage increase gradually at higher nanofiller loadings (up to 3 wt%, because of agglomeration that enable fouling attachments.

**Fig. 8:**
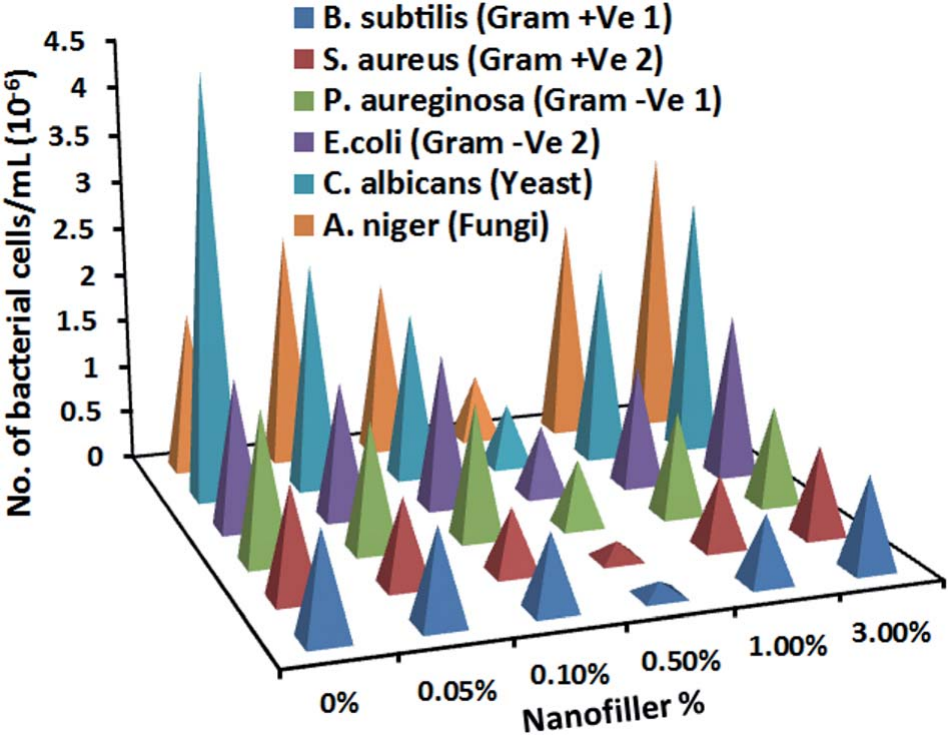
The total means of bacterial counts (cells per mL) in biofilms of the tested unfilled silicone and filled PDMS/spherical Ag@SiO_2_ core–shell nanocomposites coatings on different strains of bacteria, yeast and fungi strains after 28 days of incubation in broth media under light conditions.

**Scheme 2:**
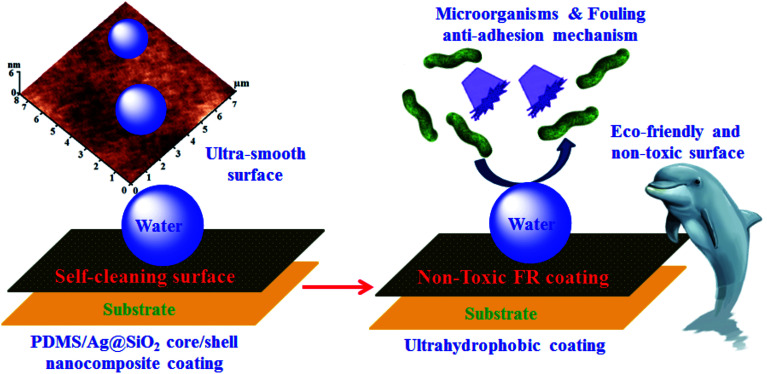
Non-toxic ultrahydrophobic surface of PDMS/spherical Ag@SiO_2_ core–shell FR nanocomposites behaviour and their failure adhesion mechanism.

Cell viability measurements (Fig. 8) reflected decreasing microorganisms' number with nanofiller insertion up to 0.5 wt%. Well-dispersion of Ag@SiO_2_ core–shell NPs results in bacterial growth inhibition by providing super-smooth and ultrahydrophobic surface and low SFE. Fouling bonds with such coating is easy to be eliminated hydrodynamically in water (Scheme 2). However at higher nanofiller loadings up to 3 wt%, microorganisms' number increases gradually because of agglomeration. This minimizes NPs' surface area and NPs/polymer interfacial bonding due to the increased van der Waals adhesion force between the particles. Also, the cell viability measurements approved higher antibacterial performance of the silicone/Ag@SiO_2_ core–shell nanocomposites over silicone/Ag hybrid film (Fig. S4†). Increased colloidal stability and dominating the distances between Ag cores within the assemblies by silica shell can prevent NP agglomeration and improve the surface area and antibacterial properties. The prepared Ag@SiO_2_ core–shell nanocomposites are more preferred in FR coatings than Ag nanospheres.

**Fig. 9:**
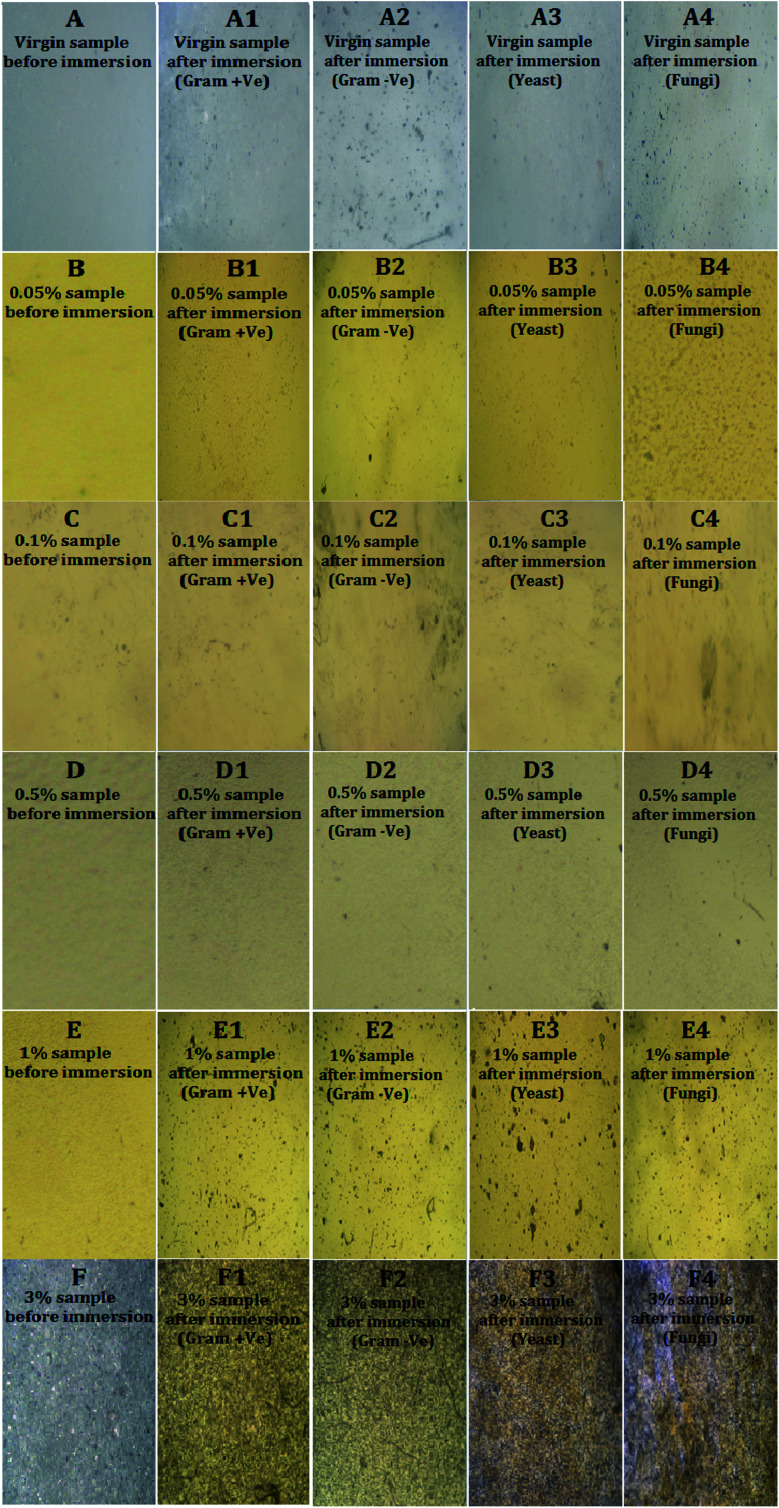
POM captures where (A), (A1), (A2), (A3) and (A4) of the virgin PDMS; (B), (B1), (B2), (B3) and (B4) of the as-synthesized PDMS/Ag@SiO_2_ core–shell nanocomposites (with 0.05% nanofiller loadings); (C), (C1), (C2), (C3) and (C4) are the images of the PDMS/Ag@SiO_2_ core–shell nanocomposites (with 0.1% nanofiller loadings); (D), (D1), (D2), (D3) and (D4) corresponding to the as-synthesized PDMS/Ag@SiO_2_ core–shell nanocomposites (with 0.5% nanofiller loadings); (E), (E1), (E2), (E3)and (E4) of the prepared PDMS/Ag@SiO_2_ core–shell nanocomposites (with 1% nanofiller loadings) and (F), (F1), (F2), (F3) and (F4) of the as-synthesized PDMS/Ag@SiO_2_ core–shell nanocomposites (with 3% nanofiller loadings) all before and after immersion in Gram (+ve and −ve) bacteria, yeast and fungi organisms for one month.

POM was used to investigate the biofilm coverage and the ability of fouling settlements on the modelled nanocomposite surface (Fig. 9). POM images approved the preparation of homogenous surface with high resistance against fouling attachments with Ag@SiO_2_ core–shell nanofiller loading up to 0.5%. On contrary, higher nano-filler loadings (up to 3 wt%) enable fouling settlement because of NPs clustering that reduced their surface area and interfacial binding with the matrix.

The antibacterial mechanism of many nanomaterials such as mesoporous nano-hexagonal Mg(OH)_2_ nanosheets and Co_3_O_4_ NPs was referred to disrupting the bacterial cell membrane and damaging DNA and cellular components. Also, it was reported that the mechanism behind the antibacterial activity of Ag NPs based on weakening DNA replication and inactivating proteins.^54,55^

However, a different mechanism was introduced here for the tailored PDMS/Ag@SiO_2_ core–shell nanocomposites based on non-toxic failure adhesion of fouling organisms. This mechanism prevents the fouling adhesion on the submerged surfaces by providing superhydrophobicity, ultra-smoothness, low SFE and self-cleaning performance of the non-leachant PDMS based coating. These factors can weaken the bonds between FR coating and fouling organisms which can be removed hydrodynamically. The high performance of well-dispersed PDMS/Ag@SiO_2_ core–shell (0.5 wt%) nanocomposites as FR coatings can be discussed as follow:

* Spherical Ag core NPs with preferential {111} growth direction that demonstration more significant antifouling properties over other silver morphologies (cubes, wires and triangular), that have fewer {111} planes.

* The polar properties Ag nanospheres with preferential {111} facets, lowest SFE per unit area and stability over the {100} and {110} facets of other morphologies, contribute to the FR and antibacterial properties.^56^

* The stability and dispersion of nano-Ag particles was enhanced through SiO_2_ shell that also improved the hydrophobicity and self-cleaning of films.^4,57^ Thus, the developed PDMS/spherical Ag@SiO_2_ core–shell nanocomposite is a promising FR coating material.

### 3.6. Evaluating the mechanical behaviour of nanocoatings

It is necessary to investigate the flexibility and surface adhesion characteristics of the modelled nanocomposite coatings. Tensile modulus was used to evaluate the nanofiller–matrix interfacial bonding and the mechanical behaviour of nanocomposites (Fig. 4B). No tensile modulus variation was observed with Ag@SiO_2_ core–shell loadings up to 0.5%, owing to the well-dispersion of nanofillers. However, at higher filling ratio up to 3 wt%, tensile modulus raised which means that the nanocomposite stiffness increased also gradually. This is the result of nanofiller aggregation that reduces the matrix–NPs interfacial bonding and causes surface heterogeneity.

The impact resistance of a polymeric material depends on the free volume available between backbone chains. The virgin PDMS and PDMS/Ag@SiO_2_ core–shell composites showed no crack in the impact test. During testing, the PDMS/Ag@SiO_2_ core–shell (0.5 wt%) composites revealed no cracks after testing up to 14 J, reflecting the flexible nature and strength caused by well-dispersed NPs (Table 1).

**Table 1:** Mechanical tests of unfilled silicone and PDMS/spherical Ag@SiO_2_ core–shell nanocomposite coatings

Properties	Concentration of PDMS/Ag@SiO_2_ core–shell nanocomposites coatings					
	0.0%	0.05%	0.10%	0.50%	1.0%	3.0%
Impact resistance (joule)	5	7	9	14	12	10
Cross-hatch	Pass	Pass	Pass	Pass	Pass	Pass
T-bending	<5	<5	<5	<5	<5	<5

Cross-hatch represents a practicable pass/fail test for evaluating the adhesion properties of the coated surface. By using cross-hatch device, almost 25–70 ideal cut places were formed, and then adhesion tape was used for testing the ruled area. In the nanocomposites, no visible adhesion defects were detected for all the specimens (Table 1).

T-bending examination technique was conducted on unfilled silicone and PDMS/Ag@SiO_2_ core–shell surface films without visible cracking for all specimens (Table 1). After identification *via* a magnifying glass, no intrusion was identified for all coated panels after bending on a <5 mm cylindrical spindle.

## 4. Conclusion

This novel work introduced an economic and ecological coating material for antifouling purposes in maritime navigation. Silver NPs were prepared *via* solvothermal method and Ag@SiO_2_ core–shell nanospheres were synthesized with 60 nm diameters and a {111} crystal plane *via* a modified Stöber method. Solution casting of silicone/spherical Ag@SiO_2_ core–shell nanocomposite surface showed fouling release *via* ultrahydrophobicity, self-cleaning effect and low surface tension. Well-dispersion of Ag@SiO_2_ core–shell nanospheres (0.5 wt% nanofillers) in silicone matrix exhibited maximum WCA and topological homogeneity; and minimum SFE and fouling adhesion. It also approved durability and tensile properties, while the viscoelastic characteristic remains unchanged. On contrarily, higher nanofiller concentrations (up to 3 wt%) induced minimum water and fouling repellency and increased SFE and topological heterogeneity caused by particle clustering. A biological assay approved lower biodegradability and cell viability of the well-dispersed nano-coatings against different bacterial strains, yeast and fungi *via* a non-stick self-cleaning technique. A comparable study approved higher WCA, lower SFE which approved higher self-cleaning properties than other commercially used FR coatings. The fabricated silicone/spherical Ag@SiO_2_ core–shell nanocomposites could possess cost-effective, long lasting properties and a green technology for shipping industry.

## Conflicts of interest

There are no conflicts to declare.

## Supplementary Material

RA-008-C8RA00351C-s001
