# Supplementary material for: Silicone/Ag@SiO2 core–shell nanocomposite as a self-cleaning antifouling coating material
Source: RSC Adv. 2018 Mar 8;8(18):9910–21. doi: 10.1039/c8ra00351c (PMC9078747; doi:10.1039/c8ra00351c)
Supplement: RA-008-C8RA00351C-s001 [file RA-008-C8RA00351C-s001.pdf]

Electronic Supplementary Material (ESI) for RSC Advances.
This journal is © The Royal Society of Chemistry 2018

Electronic Supplementary Information

Experimental

A field trial in natural sea water was performed to ensure visual assessments based on screening process and image analysis for 3 months in a tropical area. A prepared PDMS/spherical Ag@SiO_2 core-shell nanocomposite coating formulation with the nanofiller percentages 0.5 wt, (well dispersed) was used. The mixing ratio percentages were silicone (80%) and 0.5 wt. % Ag@SiO_2 core-shell nanofillers. Also, 10 wt. % ferric oxide pigments (95%, SDFCl, India) was mixed with the total formulations which were used as top coats. Test steel panels (300 mm × 200 mm × 1.5 mm) were subjected to cleaning and rust removal, then a first epoxy primer coat and epoxy-silicone tie coat layers were applied and dried. A top coat formulation of PDMS/Ag@SiO_2 core-shell nanocomposite were painted on a steel panel, left for 24 h at RT for complete drying and the thickness was 150 µm. The coated panel was tested in natural marine water at pH of 7.6–8.3, salinity of 37% and temperatures of 23–28 °C.

Results and discussion

In the current work, a novel ultrahydrophobic series of polydimethylsiloxane (PDMS)/spherical Ag@SiO_2 core/shell nanocomposites was demonstrated as fouling release (FR) paints. FTIR spectrum of Ag@SiO_2 core/shell NPs is indicated in Fig. S1a. FTIR spectrum of the PDMS was indicated in Fig. S1b. FTIR spectrum of the as-prepared PDMS/Ag@SiO_2 core-shell nanocomposite (Fig. S1c) provides evidence for the polymer-NPs interaction. The results indicated the shift in the Si–O–Si asymmetric deformation from 1098 cm^{-1} for prepared polymer to 1022 cm^{-1} for the nanocomposites. In addition, the peak for Si–O–Si skeletal stretching shifts from 802 cm^{-1} to 786 cm^{-1} for the filled specimen. Dynamic contact angle measurements including advancing, receding and hysteresis CAs were indicated in Fig. S2.

The values of S_{BET} of the prepared silicone/Ag@SiO_2 core-shell nanocomposite series were indicated in Fig. S3. Higher antibacterial performance of silicone/Ag@SiO_2 core-shell nanocomposites than silicone/Ag hybrid film was confirmed from the cell viability measurements (Fig. S4). Hybrid Ag core@SiO_2 shell nanofillers introduced potential antifouling properties than Ag NPs. Colloidal stability and the distances between Ag cores as well as the self-cleaning properties were improved by silica shell.

The biological tests were confirmed by a Field trial test of well-distributed PDMS/spherical Ag@SiO_2 core-shell nanocomposite (0.5 wt. % nanofillers, Fig. S5 (a-c)) in natural sea water for 90 days. No fouling adhesion or surface deterioration was observed for well-distributed sample (0.5 wt. % nanofillers) which confirmed the high self-cleaning and FR properties. This is caused by high WCA, low SFE and affords homogenous topology which affected by well-dispersion of Ag@SiO_2 core-shell nanospheres fillers.

ESI Figures:

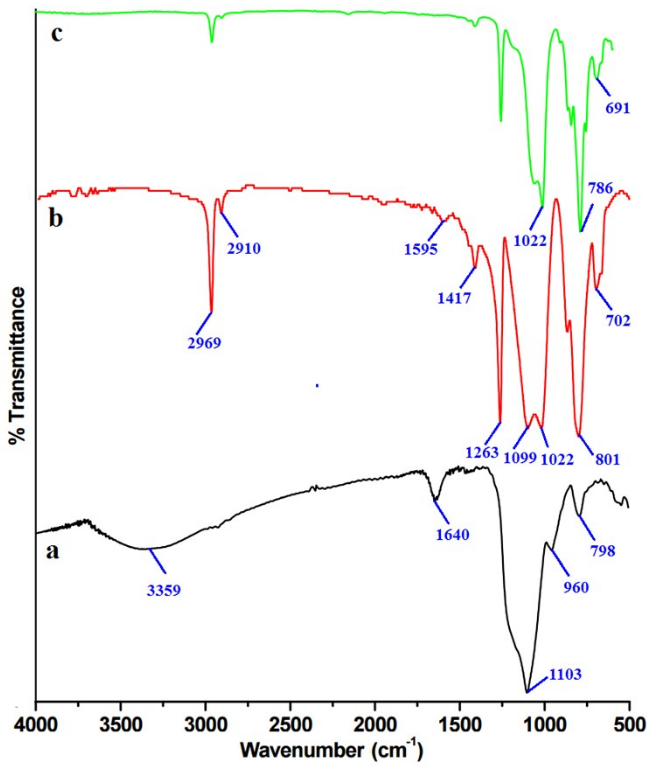

Fig. S1: FTIR spectra of (a) Ag@SiO_2 core-shell particles; (b) the as-prepared PDMS; and (c) PDMS/Ag@SiO_2 core-shell nanocomposites.

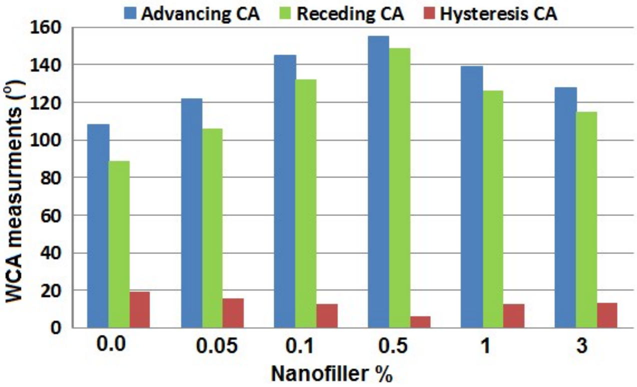

Fig. S2: Dynamic water contact angle measurements: advancing, receding and hysteresis angle of the prepared PDMS/Ag@SiO_2 core-shell nanocomposites (Error bars represent ±2 standard deviations from three replications).

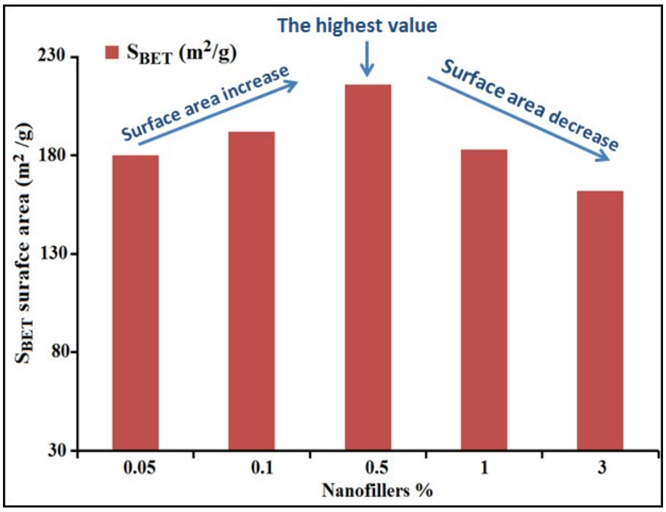

Fig. S3: S_{BET} surface area of PDMS/Ag@SiO_2 core-shell nanocomposites.

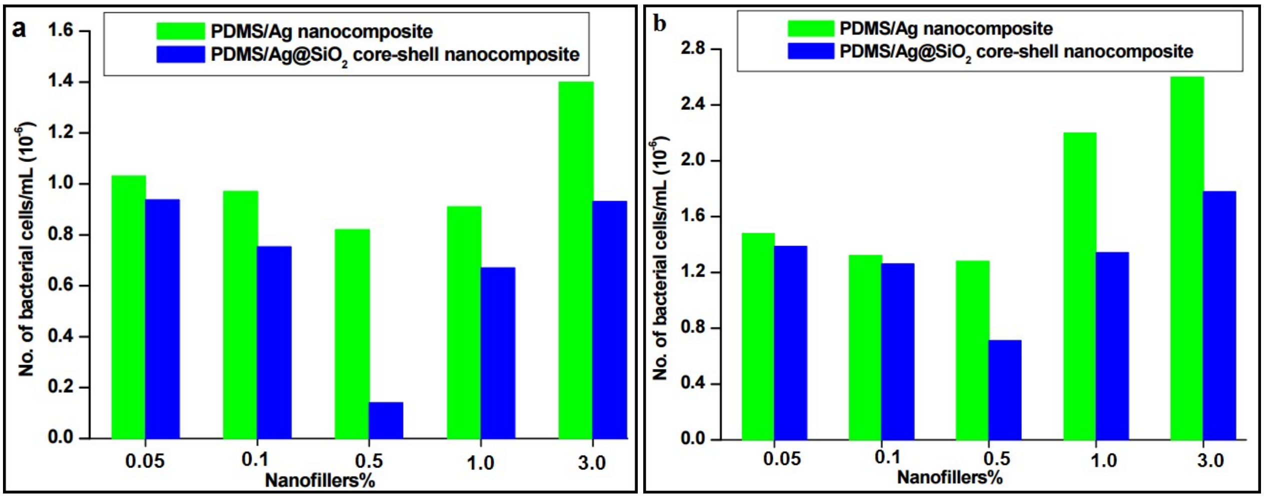

Fig. S4: The total means of bacterial counts (cells/ml) in biofilms of the silicone/Ag nanocomposites and silicone/Ag@SiO_2 core-shell nanocomposite coatings using (a) B. subtilis (Gram +Ve) and (b) E.coli (Gram -Ve) bacteria incubation in broth media under light conditions.

This journal is © The Royal Society of Chemistry 2015

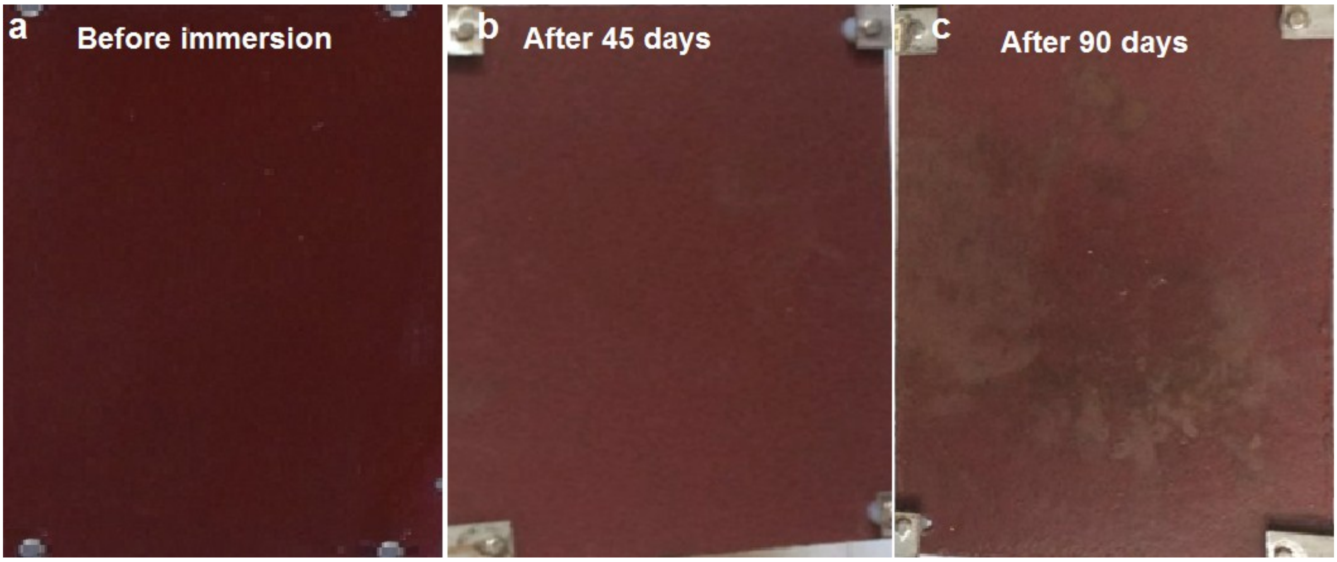

Fig. S5: (a)–(c) Field test results of the formulated PDMS/spherical $Ag@SiO_2$ core-shell (0.5 wt. % nanofiller) composite coating after 90 days of immersion in natural sea water.
